# Suppression of Dynamical Network Biomarker Signals at the Predisease State (*Mibyou*) before Metabolic Syndrome in Mice by a Traditional Japanese Medicine (Kampo Formula) Bofutsushosan

**DOI:** 10.1155/2020/9129134

**Published:** 2020-08-04

**Authors:** Keiichi Koizumi, Makito Oku, Shusaku Hayashi, Akiko Inujima, Naotoshi Shibahara, Luonan Chen, Yoshiko Igarashi, Kazuyuki Tobe, Shigeru Saito, Makoto Kadowaki, Kazuyuki Aihara

**Affiliations:** ^1^Division of Kampo Diagnostics, Institute of Natural Medicine, University of Toyama, Toyama, Japan; ^2^Laboratory of Drug Discovery and Development for Pre-disease, Section of Host Defences, Division of Bioscience, Institute of Natural Medicine, University of Toyama, Toyama, Japan; ^3^Division of Chemo-Bioinformatics, Institute of Natural Medicine, University of Toyama, Toyama, Japan; ^4^Laboratory of Chemo-Bioinformatics, Section of Host Defences, Division of Bioscience, Institute of Natural Medicine, University of Toyama, Toyama, Japan; ^5^Division of Gastrointestinal Pathophysiology, Institute of Natural Medicine, University of Toyama, Toyama, Japan; ^6^Laboratory of Gastrointestinal Disorder, Section of Host Defences, Division of Bioscience, Institute of Natural Medicine, University of Toyama, Toyama, Japan; ^7^CAS Center for Excellence in Molecular Cell Science, Institute of Biochemistry and Cell Biology, Shanghai Institutes for Biological Sciences, Chinese Academy of Sciences, Shanghai, China; ^8^Institute of Industrial Science, The University of Tokyo, Tokyo, Japan; ^9^First Department of Internal Medicine, Faculty of Medicine, University of Toyama, Toyama, Japan; ^10^University of Toyama, Toyama, Japan; ^11^International Research Center for Neurointelligence (WPI-IRCN), The University of Tokyo Institutes for Preemptive Study, The University of Tokyo, Tokyo, Japan

## Abstract

Due to the increasing incidence of metabolic syndrome, the development of new therapeutic strategies is urgently required. One promising approach is to focus on the predisease state (so-called *Mibyou* in traditional Japanese medicine) before metabolic syndrome as a preemptive medical target. We recently succeeded in detecting a predisease state before metabolic syndrome using a mathematical theory called the dynamical network biomarker (DNB) theory. The detected predisease state was characterized by 147 DNB genes among a total of 24,217 genes in TSOD (Tsumura-Suzuki Obese Diabetes) mice, a well-accepted model of metabolic syndrome, at 5 weeks of age. The timing of the predisease state was much earlier than the onset of metabolic syndrome in TSOD mice reported to be at approximately 8–12 weeks of age. In the present study, we investigated whether the predisease state in TSOD mice can be inhibited by the oral administration of a Kampo formula, bofutsushosan (BTS), which is usually used to treat obese patients with metabolic syndrome in Japan, from 3 to 7 weeks of age. We found the comprehensive suppression of the early warning signals of the DNB genes by BTS at 5 weeks of age and later. Specifically, the standard deviations of 134 genes among the 147 DNB genes decreased at 5 weeks of age as compared to the nontreatment control group, and 80 of them showed more than 50% reduction. In addition, at 7 weeks of age, the body weight and blood glucose level were significantly lower in the BTS-treated group than in the nontreatment control group. The results of our study suggest a novel mechanism of BTS; it suppressed fluctuations of the DNB genes at the predisease state before metabolic syndrome and thus prevented the subsequent transition to metabolic syndrome. In conclusion, this study demonstrated the preventive and preemptive effects of a Kampo formula on *Mibyou* before metabolic syndrome for the first time based on scientific evaluation.

## 1. Introduction

The incidence of obesity is markedly increasing worldwide. The World Health Organization (WHO) reported that more than 650 million (13%) adults were obese in 2016, and the obesity rate has increased nearly three-fold since 1975 [1]. Because obesity is a major cause of metabolic syndrome, the incidence of metabolic syndrome is also increasing considerably, which causes the increased risk for lifestyle-related diseases [2–4]. Therefore, the development of new therapeutic strategies for metabolic syndrome is needed. Clues for such strategies are provided in the oldest known medical text, *Huang Di Nei Jing* (Yellow Emperor's Inner Canon) [5], which was written more than 2,000 years ago in China. The text states that the best therapeutic strategy is to cure the predisease state (so-called *Mibyou* [6] in traditional Japanese medicine or *Wei Bing* [5] in traditional Chinese medicine). Therefore, it is a promising approach to focus on the predisease state before metabolic syndrome as a preemptive medical target.

In our most recent study [7], we succeeded in detecting a predisease state before metabolic syndrome in mice by using a mathematical theory called the dynamical network biomarker (DNB) theory [8]. The DNB theory has been established based on the critical transition paradigm [9] in order to detect the predisease state objectively and quantitatively [8]. We applied the DNB theory to the metabolic syndrome model mice called Tsumura-Suzuki Obese Diabetes (TSOD) mice [10, 11] that have been known to develop a wide range of disorders similar to human metabolic syndrome including hyperglycemia [11], hypertension [12], dyslipidemia [11], glucose intolerance [10], insulin resistance [13], peripheral neuropathy [14], intestinal dysbiosis [15], and nonalcoholic fatty liver disease [16]. We revealed that the predisease state was characterized by elevated fluctuations and strengthened correlations of the 147 DNB genes among a total of 24,217 genes [7]. We detected the predisease state in TSOD mice at 5 weeks of age, whose timing was much earlier than the onset of metabolic syndrome in TSOD mice reported to be at approximately 8–12 weeks of age [13, 16, 17].

On the other hand, many traditional Japanese medicines (Kampo formulas) are empirically believed to be effective not only during the disease state but also during the predisease state. Traditional Japanese medicine was derived from traditional Chinese medicine and has been developed independently in Japan. In the practice of traditional Japanese medicine, treatment at the predisease state (*Mibyou* in Japanese) has long been emphasized. Terasawa et al. defined *Mibyou* as “disease-oriented state: not a disease, but can easily become one if no cure is applied” [6]. However, therapeutic effects of Kampo formulas against the predisease state have not been scientifically validated.

**Figure 1:**
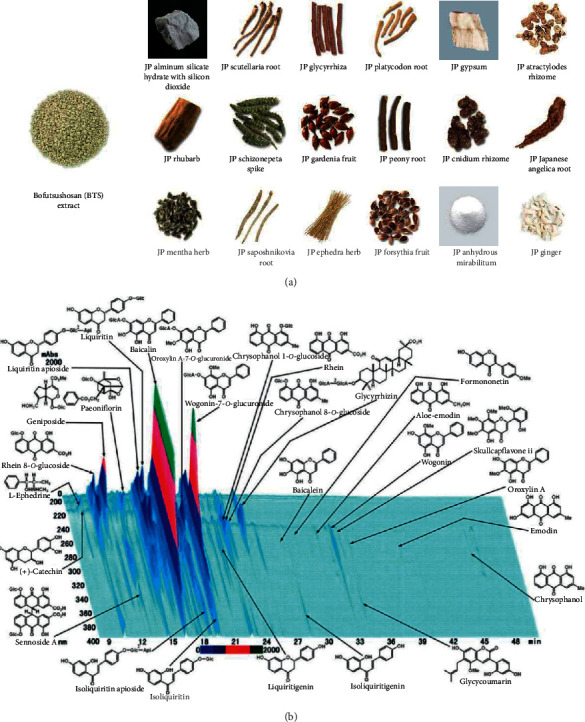
A Kampo formula BTS. (a) Pictures of BTS extract and its herb and mineral compositions. (b) PDA-HPLC profiles of the BTS extract used in the present study. JP: Japanese Pharmacopoeia. These figures were provided by Tsumura & Co.

Therefore, in the present study, we attempted to reveal whether the emergence of the predisease state before metabolic syndrome in TSOD mice, which was characterized by the 147 DNB genes, can be prevented by the oral administration of a Kampo formula. We used bofutsushosan (BTS), a Kampo formula composed of 18 types of herbs and minerals (Figure 1 and Table 1), which is widely used in the treatment of obese patients with metabolic syndrome in Japan. In randomized controlled clinical trials, BTS significantly decreased body weights [18, 19], reduced visceral fat masses [18], improved insulin resistance [18], and suppressed ambulatory blood pressure variability [20]. In TSOD mice, BTS ameliorates various symptoms of metabolic syndrome, such as body weight gain, visceral/subcutaneous fat accumulation, abnormal glucose tolerance, elevation of blood pressure, peripheral neuropathy, and so on [12]. Although the safety and efficacy of BTS are supported by evidence, its mechanism of action has not been elucidated because of the large number of natural compounds it contains (Figure 1(b)). This is the first study to demonstrate the possibility of preventive and preemptive treatment targeting the predisease state (*Mibyou*) before metabolic syndrome by a Kampo formula.

## 2. Experimental Procedures

### 2.1. Kampo Formula BTS

BTS was obtained from Tsumura & Co. (Tokyo, Japan). BTS was prepared as a spray-dried powder of a hot water extract obtained from eighteen medicinal herbs and minerals (Figure 1) in the ratio listed in Table 1. BTS extract has been approved by the Japanese Ministry of Health, Labour and Welfare. It is defined in the Japanese Pharmacopoeia 17^th^ edition (JP 17), which is established and published to regulate the properties and quality of drugs by the Japanese Ministry of Health, Labour and Welfare after hearing the opinion of the Pharmaceutical Affairs and Food Sanitation Council. High-performance liquid chromatography (HPLC) analyses of the ingredients of BTS were performed using photodiode array (PDA)-HPLC by Tsumura & Co. The PDA-HPLC profiles of the samples are shown in Figure 1.

### 2.2. Spontaneous Mouse Model of Metabolic Syndrome

Three-week-old male TSOD (*n* = 39) mice were purchased from the Institute for Animal Reproduction (Ibaraki, Japan). Mice were housed in groups of two or three per cage, maintained in a specific pathogen free (SPF) condition at 24 ± 2°C on a 12-hour light and 12-hour dark cycle, and given normal chow diet (MF; Oriental Yeast Co., Ltd., Tokyo, Japan) and water ad libitum. The mice were assigned to the control (untreated) and the BTS-treated group (Table 2 and Supplementary Figure 1) so that each group had similar weights. In the BTS-treated group, BTS was administrated to TSOD mice (1000 mg/kg body weight/day, per os, at 10 a.m.) every day. Nonfasting blood glucose concentrations in tail vein blood and body weights were measured in 3-, 4-, 5-, 6-, and 7-week-old TSOD mice. After measurements, the mice were dissected to collect epididymal white adipose tissue under anesthesia to minimize suffering. Regarding each adipose tissue sample of individual mice, the total RNA was extracted using the RNeasy Total RNA Extraction kit (Qiagen, Valencia, CA, USA). The numbers of samples [7] taken for subsequent analyses are shown in Table 2. This animal study was performed in strict accordance with the recommendations in the Guide for the Care and Use of Laboratory Animals of University of Toyama. The protocol was approved by the Committee on the Ethics of Animal Experiments of University of Toyama (Permit Number: A2014INM-11). All surgery was performed under optimal anesthesia (a combination anesthetic with medetomidine (0.3 mg/kg), midazolam (4.0 mg/kg), and butorphanol (5.0 mg/kg)), and all efforts were made to minimize suffering. The animals were humanely sacrificed by cervical dislocation under the combination anesthesia.

### 2.3. Microarray Assay

In order to investigate the gene expression profiles of each mouse, the Agilent SurePrint G3 Mouse Gene Expression 8 × 60K Microarray Kit (Agilent Technologies, Santa Clara, CA, USA) was used. Cyanine-3- (Cy3-) labeled cRNA was prepared from 0.1 *μ*g total RNA using the Low Input Quick Amp Labeling Kit (Agilent) according to the manufacturer's instructions, followed by RNeasy column purification (QIAGEN, Valencia, CA). Dye incorporation and cRNA yield were checked with the NanoDrop ND-2000 Spectrophotometer (Thermo Fisher Scientific, Waltham, MA, USA) and Agilent 2100 Bioanalyzer (Agilent). A total of 0.6 *μ*g of Cy3-labeled cRNA was fragmented at 60°C for 30 minutes in a reaction volume of 25 *μ*l containing 1 × Agilent fragmentation buffer and 2 × Agilent blocking agent following the manufacturer's instructions. On completion of the fragmentation reaction, 25 *μ*l of 2 × Agilent hybridization buffer was added to the fragmentation mixture and hybridized to SurePrint G3 Mouse Gene Expression 8 × 60K Microarray (Agilent) at 65°C for 17 hours in a rotating Agilent hybridization oven. After hybridization, microarrays were washed at room temperature for 1 minute with GE Wash Buffer 1 (Agilent) and at 37°C for 1 minute with GE Wash buffer 2 (Agilent). Slides were scanned immediately after washing on the Agilent G2505C Microarray Scanner System (Agilent) using the one color scan setting for 8 × 60k array slides (scan area 61 mm × 21.6 mm, scan resolution 3 *μ*m, the dye channel was set to green, and photomultiplier tube (PMT) gain was set to 100%). Scanned images were analyzed with Feature Extraction Software 12.0.3.1 (Agilent) using default parameters to obtain background-subtracted and spatially detrended processed signal intensities.

### 2.4. Microarray Data Preprocessing

The probe names in the raw dataset were converted to gene symbols according to the annotation table used in our previous work [7]. Probe names without any gene symbol annotation were removed. When multiple probe names were assigned to a single gene symbol, the mean value was taken. Gene expression values were then divided by the 2% trimmed mean (the mean value calculated by discarding the lowest 2% and highest 2% values) in each sample in order to normalize the dataset. Normalized values were base-2 log-transformed.

### 2.5. Extraction of Differentially Expressed Genes

Differentially expressed genes (DEGs) are genes with expression values that markedly change between different conditions or groups. In the present study, DEGs were extracted based on fold-changes and hypothesis testing. In fold change filtering, the arithmetic mean of the log-transformed values (or equivalently, the logarithm of the geometric mean in the original scale) was calculated for each gene in each group. Genes that exhibited more than one intergroup difference, which corresponded to more than a two-fold change in the original scale, were taken as the first group of DEG candidates. On the other hand, two-tailed Welch's *t*-tests were performed for each gene using log-transformed values. In order to alleviate the large risk of Type-I errors in multiple hypothesis testing, we adjusted the significance level by the Benjamini–Hochberg (BH) procedure [21], which controls the supremum of the expected value of the false discovery rate (FDR). The genes for which the null hypothesis was rejected based on the adjusted significance level (E(FDR) ≤0.05) were taken as the second group of DEG candidates. We extracted the intersection of the two candidate groups as DEGs.

### 2.6. Clustering Analysis

We used a hierarchical clustering method to find gene clusters that showed similar time evolutions. Since the dynamic range of the gene expression profiles was large, we initially performed z-score normalization for each gene. Dissimilarity between genes was evaluated based on 1-*r*_*ij*_, where *r*_*ij*_ is the correlation coefficient between the *i*th and *j*th genes. The average linkage method was then used to calculate the dendrogram. Genes were separated into clusters with a cutoff value of 0.5 for intercluster dissimilarity.

### 2.7. Enrichment Analysis

Enrichment analyses of GO annotations and the KEGG pathways were performed using the web tool of the DAVID (Database for Annotation, Visualization and Integrated Discovery) database [22] (https://david.ncifcrf.gov). We initially picked up all gene sets with hit counts greater than or equal to 2, regardless of *p* values. We then calculated *p* values based on Fisher's exact test. Next, the BH procedure was used to control FDR below 0.05. Finally, top 10 gene sets were selected based on the hit counts. It was confirmed that similar results could be obtained by correcting *p* values by another method adopted in the DAVID database.

### 2.8. DNB Scores

In order to quantify the effects of BTS on the fluctuations and correlations among the DNB genes that were identified in our previous study, we calculated two DNB scores, the average standard deviation *I*_s_ and the average correlation strength *I*_r_, which are defined as follows:

(1) $$\begin{array}{c} I_{\text{s}}=\frac{1}{\left|S\right|}\sum_{i\in S}s_{i},\\ I_{\text{r}}=\frac{2}{\left|S\right|\left(\left|S\right|-1\right)}\sum_{i,j\in S,\;i<j}\left|r_{ij}\right|-c, \end{array}$$

where *S* is the indices of the DNB genes, |*S*| denotes the number of the DNB genes, *s*_*i*_ is the standard deviation of gene *i*, *r*_*ij*_ is the correlation coefficient between gene *i* and gene *j*, and *c* is a correction term that depends on the sample size (*c* = 0.64, 0.50, and 0.42 for 3, 4, and 5 samples, respectively). The correction term represents the expected correlation strength of two independent random variables both following the standard normal distribution. We introduced this term because a systematic increase in correlation strength was observed in subdata with only 3 or 4 samples.

### 2.9. Network Analysis

The network analysis was performed mainly in the web tool of the STRING (Search Tool for the Retrieval of Interacting Genes/Proteins) database [23, 24] (https://string-db.org). The STRING database provides protein-protein interaction (PPI) networks based on multiple resources, such as experiments, gene coexpression, and text-mining. By integrating evidence from multiple resources, the combined scores between 0 and 1 are calculated and assigned to each edge of the PPI networks [24]. A larger combined score indicates that the corresponding interaction is supported by a large amount of strong evidence. It is important to note that the combined scores do not represent the strengths of chemical affinity. We uploaded the union set of the DEGs and DNBs to the web tool of the STRING v10.0 database [23] and obtained the induced subgraph with combined scores of more than 0.4, which was the default setting in the web tool. The result was downloaded and processed further by the NetworkX package of python to remove small connected components with only one or two nodes. The result was visualized by Graphviz, a graph visualization software.

## 3. Results

**Figure 2:**
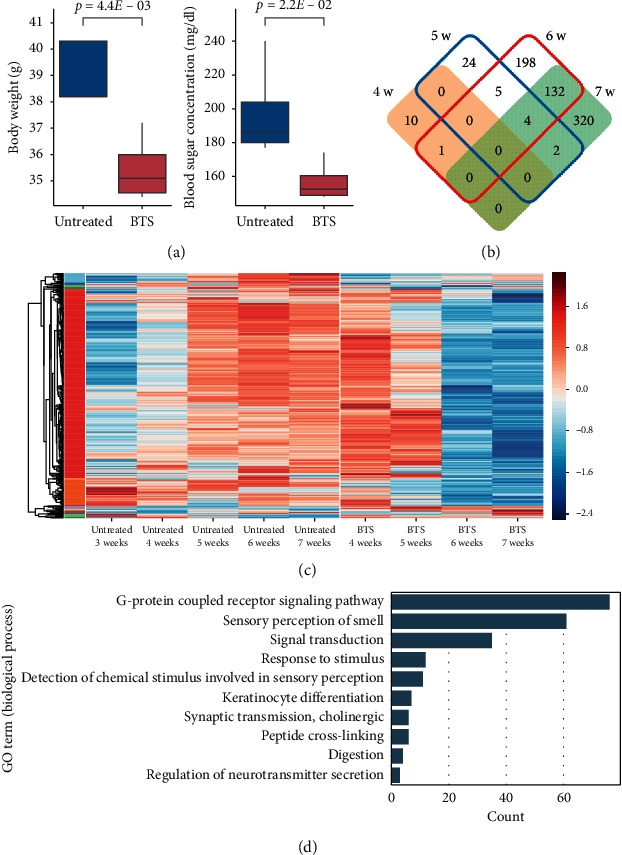
Effects of BTS on TSOD mice. (a) Box plots of body weights and blood sugar concentrations at 7 weeks of age. In the left most box, the median line matched the box bottom. *p* values were based on the two-tailed Welch's *t*-test. (b) Venn diagram of four DEG sets. (c) Heatmap of the union set of the four DEG sets. The color scale shows the z-score (per row) of the log expression. (d) GO annotations enriched in the largest cluster. A KEGG pathway “Olfactory transduction” was also enriched in the cluster (59 genes overlapping).

We compared the BTS-treated group, wherein BTS was orally administered every day from 3 weeks of age, with the nontreatment control group (Supplementary Figure 1). The numbers of analyzed samples are shown in Table 2. An adverse event was not observed on the experimental mice in this study. The body weight and blood glucose level at 7 weeks of age were significantly lower in the BTS-treated group than those in the control group, as shown in Figure 2(a). This confirmed that there were observable differences between the BTS-treated and untreated groups.

We then compared the gene expression profiles of the two groups and obtained DEGs at each time period (Figure 2(b) and Supplementary Figure 1). The numbers of DEGs were 11, 35, 340, and 458 for 4, 5, 6, and 7 weeks of age, respectively. This number suddenly increased at 6 weeks of age and remained high at 7 weeks of age, which indicated that a marked deviation between the two groups occurred from 6 weeks of age in the gene expression profiles. We then performed a clustering analysis of the union set of the four DEG sets (696 genes) and found that most genes showed a similar time evolution and formed a huge cluster comprising 537 genes (Figure 2(c)). The largest cluster was associated with several biological functions, including signal transduction, olfactory perception, and keratinocyte differentiation (Figure 2(d)). Such diverse effects of BTS were expected because it contains many natural compounds extracted from 18 crude drugs. The 696 DEGs included only six DNB genes (*March10*, *Oxct2a*, *Stpg4*, *Tuba3b*, *1700061N14Rik*, and *4933406F09Rik*), all of which were in the largest cluster. The size of the overlap was close to the chance level (4.2 genes) and not significant (*p* = 0.25, Fisher's exact test). This result suggested that the administration of BTS did not have a significant influence on the average expression levels of most DNB genes. In addition, neither GO annotation nor the KEGG pathway was enriched in the six genes.

**Figure 3:**
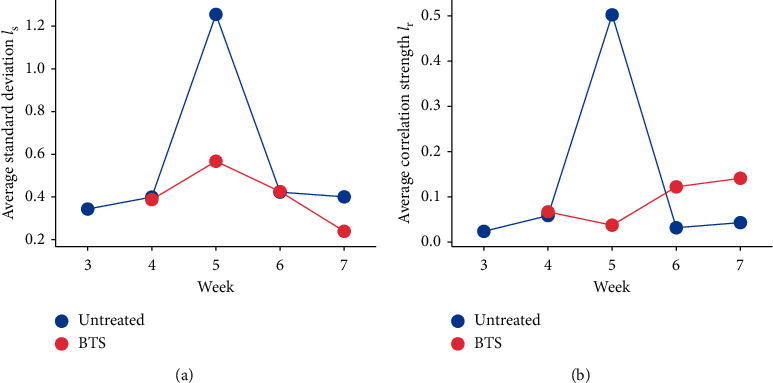
Effects of BTS on DNB scores. (a) Average standard deviation *I*_s_. (b) Average correlation strength *I*_r_.

On the other hand, the peaks of the DNB scores at 5 weeks of age disappeared almost completely in the BTS-treated group (Figures 3(a) and 3(b)). In other words, BTS suppressed the emergence of unusual fluctuations and correlations in the DNB genes at the putative predisease state before metabolic syndrome. Specifically, the standard deviations of 134 genes among the 147 DNB genes decreased at 5 weeks of age as compared to the nontreatment control group, and 80 of them showed more than 50% reduction. The average standard deviation *I*_s_ and the average correlation strength *I*_r_ decreased to 45% and 7.4% of those of the untreated group, respectively.

**Figure 4:**
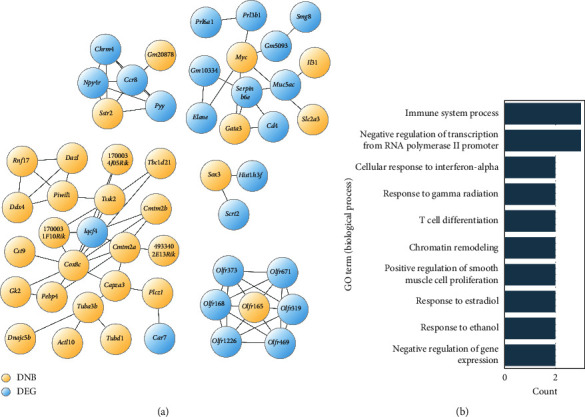
Interactions between the DNB genes and DEGs regulated by BTS. (a) Interactions between the DNB genes (yellow) and DEGs regulated by BTS after 6 weeks of age (blue). (b) GO annotations enriched in the upper right subcluster of Figure 4(a). A KEGG pathway “Transcriptional misregulation in cancer” was also enriched in it with 2 genes overlapping.

We further investigated interactions between the DNB genes and DEGs based on a conjecture that the fluctuated DNB genes at 5 weeks of age in the nontreatment group may have served as the driving force of DEGs appearing after 6 weeks age. We initially extracted 123 genes that were expressed differentially at 6 and 7 weeks of age from the largest cluster. The selected DEGs did not include any DNB gene. Known or predicted interactions between the DNB genes and selected DEGs were then searched in the STRING database [23]. As a result, we found 5 subclusters with which the main DNB genes and DEGs were closely related (Figure 4(a)). In the upper right subcluster in Figure 4(a), more than three genes participated from each group. The GO enrichment analysis of the subcluster predicted the involvement of immune response functions (Figure 4(b)). Enriched GO annotations were assigned to *Gata3*, *Myc*, *Cd4*, *Elane*, and *Il31*.

## 4. Discussion

In this study, we attempted to reveal how a Kampo formula BTS affects the metabolic syndrome model mouse, TSOD mouse, particularly at the predisease state [7]. Our results demonstrated the preventive and preemptive effects of BTS on the predisease state before metabolic syndrome, which were scientifically quantified by the DNB theory. Major contribution of the present study is to show that the BTS administration comprehensively suppressed the fluctuations and correlations of the 147 DNB genes in TSOD mice at 5 weeks of age (Figure 3), suggesting therapeutic effects of BTS on the predisease state before metabolic syndrome. Because increases in fluctuations and correlation strength generally indicate reduced stability or resilience from the viewpoint of dynamical systems theory [8, 9, 25, 26], the elimination of the peaks suggests that BTS helps to retain high stability or resilience in the health condition of TSOD mice, which may, in turn, prevent abrupt disease progression. Our results may lead to a new research direction based on the suppression of the DNB scores. It will provide additional understanding of the efficacy of BTS and other Kampo formulas that are empirically believed to be effective for curing the predisease state. In addition, the DNB scores will help to quantify how lifestyle changes regarding diet and exercise habits improve the stability of health conditions and thus contribute to facilitate preventive and preemptive therapy targeting *Mibyou*.

Regarding biological interest, we have identified the five genes *Gata3* [27], *Myc* [28], *Cd4* [29], *Elane* [30], and *Il31* [31] as putative key regulators at the predisease state before metabolic syndrome based on DNB-DEG relationships (Figure 4). *Gata3* encodes the transcription factor GATA-3, which is a master regulator of T helper 2 (Th2) cells. The transcription factor MYC encoded by *Myc* is well-known for its role in tumorigenesis, but it also regulates a broad range of genes, including those associated with immune responses. CD4 encoded by *Cd4* is a surface marker of T helper cells. *Elane* encodes neutrophil elastase, a neutrophil-releasing protease. Interleukin-31 encoded by *Il31* is a proinflammatory cytokine released by CD4+ T helper cells. All five genes are associated with the immune system. Although immune responses and inflammation are well-known key factors of the pathogenesis of metabolic syndrome, currently, only limited information is available on the roles of these genes, particularly at the predisease state (*Mibyou*) or subsequent transition from a healthy state to metabolic syndrome. We will attempt to clarify their roles in details, which will lead to discovery of novel molecular therapeutic targets as well as establishment of reliable biomarkers for metabolic syndrome.

The results shown in Figure 3 have provided clues for understanding how BTS works in terms of the DNB theory. The DNB theory, in its simplest version, assumes the existence of three states: the healthy state, predisease (*Mibyou*) state, and disease state. The DNB theory can be generalized to the cases where multiple transitions occur during disease progression. We argued in our previous study [7] that the untreated TSOD mice were initially at the healthy state, then the predisease state appeared at 5 weeks of age, and finally, the mice settled into another stable state, one step closer to metabolic syndrome. In contrast, the present study showed that the peaks of the DNB scores at 5 weeks of age disappeared in the BTS-treated TSOD mice (Figure 3). Therefore, we consider that BTS prevented entry into the predisease state or eliminated the presence of the predisease state.

## 5. Conclusion

This is the first study to demonstrate objectively and quantitatively that a Kampo formula has therapeutic effects on the predisease state (*Mibyou*). In contrast to Kampo formulas that have been empirically believed to have therapeutic effects on the predisease state, many modern medical drugs have been designed to target for symptoms at the disease state, such as hyperglycemia in metabolic syndrome [32]. Based on marked increases in the incidence of complex diseases, such as metabolic syndrome, we cannot treat them using only modern medical drugs. In contrast to the symptomatic treatments by modern medical drugs at the disease state, Kampo formulas targeting the predisease state may have great potential advantages as preventive and preemptive medicine [33–36]. We expect that such a *Mibyou*-targeting approach will be established by the progression of interdisciplinary research of mathematics and traditional medicine.

## Figures and Tables

**Table 1:** Composition of BTS extract granules for ethical use (TJ-62) employed in the present study.

Crude drug name	Amount (g)
JP aluminum silicate hydrate with silicon dioxide	3.0
JP scutellaria root	2.0
JP glycyrrhiza	2.0
JP platycodon root	2.0
JP gypsum	2.0
JP atractylodes rhizome	2.0
JP rhubarb	1.5
JP schizonepeta spike	1.2
JP gardenia fruit	1.2
JP peony root	1.2
JP cnidium rhizome	1.2
JP Japanese angelica root	1.2
JP mentha herb	1.2
JP saposhnikovia root	1.2
JP Ephedra herb	1.2
JP forsythia fruit	1.2
JP anhydrous mirabilitum	0.7
JP ginger	0.3

A total of 4.5 g of a dried extract was obtained from the above described mixed crude drugs. By adding inactive ingredients (JP light anhydrous silicic acid, JP magnesium stearate, and JP lactose hydrate), 7.5 g of the extract granules was prepared. JP: Japanese pharmacopoeia.

**Table 2:** The numbers of analyzed samples. Samples with insufficient quality for microarray assay were excluded.

Group	3 weeks	4 weeks	5 weeks	6 weeks	7 weeks
Control (untreated)	5	5	5	4	5
BTS-treated	—	3	4	4	4

## Data Availability

Source codes used in the present study for statistical analyses will be made available in the GitHub repository under MIT license (https://github.com/okumakito/dnb-bts). The microarray datasets generated and analyzed during the present study are available in the Gene Expression Omnibus (GEO) repository through GEO series accession number GSE112653 (https://www.ncbi.nlm.nih.gov/geo/query/acc.cgi?acc=GSE112653).

## Supplementary Materials

Supplementary Materials Supplementary Figure 1: schematic of the experimental design. The four arrows labeled 528 DEGs indicate the comparison made to extract DEGs.
