# Corrigendum to “Suppression of Dynamical Network Biomarker Signals at the Predisease State (*Mibyou*) before Metabolic Syndrome in Mice by a Traditional Japanese Medicine (Kampo Formula) Bofutsushosan”

**DOI:** 10.1155/2021/9760307

**Published:** 2021-04-22

**Authors:** Keiichi Koizumi, Makito Oku, Shusaku Hayashi, Akiko Inujima, Naotoshi Shibahara, Luonan Chen, Yoshiko Igarashi, Kazuyuki Tobe, Shigeru Saito, Makoto Kadowaki, Kazuyuki Aihara

**Affiliations:** ^1^Division of Kampo Diagnostics, Institute of Natural Medicine, University of Toyama, Toyama, Japan; ^2^Laboratory of Drug Discovery and Development for Pre-disease, Section of Host Defences, Division of Bioscience, Institute of Natural Medicine, University of Toyama, Toyama, Japan; ^3^Division of Chemo-Bioinformatics, Institute of Natural Medicine, University of Toyama, Toyama, Japan; ^4^Laboratory of Chemo-Bioinformatics, Section of Host Defences, Division of Bioscience, Institute of Natural Medicine, University of Toyama, Toyama, Japan; ^5^Division of Gastrointestinal Pathophysiology, Institute of Natural Medicine, University of Toyama, Toyama, Japan; ^6^Laboratory of Gastrointestinal Disorder, Section of Host Defences, Division of Bioscience, Institute of Natural Medicine, University of Toyama, Toyama, Japan; ^7^CAS Center for Excellence in Molecular Cell Science, Institute of Biochemistry and Cell Biology, Shanghai Institutes for Biological Sciences, Chinese Academy of Sciences, Shanghai, China; ^8^Institute of Industrial Science, The University of Tokyo, Tokyo, Japan; ^9^First Department of Internal Medicine, Faculty of Medicine, University of Toyama, Toyama, Japan; ^10^University of Toyama, Toyama, Japan; ^11^International Research Center for Neurointelligence (WPI-IRCN), The University of Tokyo Institutes for Preemptive Study, The University of Tokyo, Tokyo, Japan

In the article titled “Suppression of Dynamical Network Biomarker Signals at the Predisease State (*Mibyou*) before Metabolic Syndrome in Mice by a Traditional Japanese Medicine (Kampo Formula) Bofutsushosan” [1], the authors wish to clarify that the DNB scores of the untreated group shown in blue in Figure 3 were recalculated from data obtained from the Gene Expression Omnibus (GEO) repository, accession number: GSE112653 [2], and were presented for comparison. The authors confirm that this does not affect the results and conclusions of the article.
