# Supplementary figures and images for: Suppression of Dynamical Network Biomarker Signals at the Predisease State (Mibyou) before Metabolic Syndrome in Mice by a Traditional Japanese Medicine (Kampo Formula) Bofutsushosan
Source: Evid Based Complement Alternat Med. 2020 Aug 4;2020:9129134. doi: 10.1155/2020/9129134 (PMC7424500; doi:10.1155/2020/9129134)

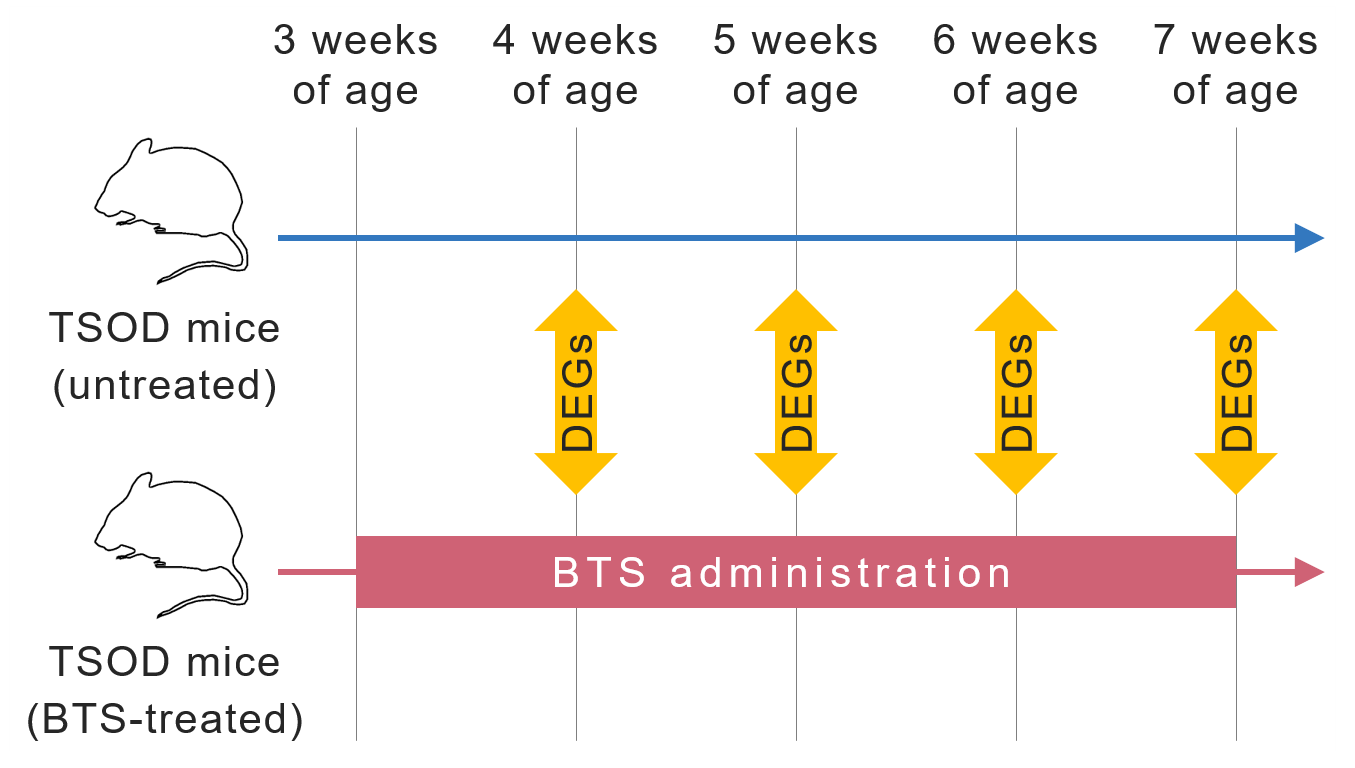

Supplement: Supplementary Materials — Supplementary Figure 1: schematic of the experimental design. The four arrows labeled 528 DEGs indicate the comparison made to extract DEGs. [file 9129134.f1.png]
